# Cardiac Ischemia On-a-Chip: Antiarrhythmic Effect of Levosimendan on Ischemic Human-Induced Pluripotent Stem Cell-Derived Cardiomyocytes

**DOI:** 10.3390/cells11061045

**Published:** 2022-03-19

**Authors:** Mahmoud Gaballah, Kirsi Penttinen, Joose Kreutzer, Antti-Juhana Mäki, Pasi Kallio, Katriina Aalto-Setälä

**Affiliations:** 1 Heart Group, Faculty of Medicine and Health Technology, Tampere University, 33520 Tampere, Finland; kirsi.penttinen@outlook.com (K.P.); katriina.aalto-setala@tuni.fi (K.A.-S.); 2 Department of Forensic Medicine and Toxicology, Faculty of Veterinary Medicine, University of Sadat City, Menoufia 32897, Egypt; 3 Micro- and Nanosystems Research Group, Faculty of Medicine and Health Technology, Tampere University, 33520 Tampere, Finland; joose.kreutzer@tuni.fi (J.K.); antti-juhana.maki@tuni.fi (A.-J.M.); pasi.kallio@tuni.fi (P.K.); 4 Heart Hospital, Tampere University Hospital, 33520 Tampere, Finland

**Keywords:** ischemic heart disease, cardiac ischemia on-a-chip, human-induced pluripotent stem cell-derived cardiomyocytes, calcium transient, levosimendan, antiarrhythmic effect

## Abstract

Ischemic heart disease (IHD) is one of the leading causes of mortality worldwide. Preserving functionality and preventing arrhythmias of the heart are key principles in the management of patients with IHD. Levosimendan, a unique calcium (Ca^2+^) enhancer with inotropic activity, has been introduced into clinical usage for heart failure treatment. Human-induced pluripotent cell-derived cardiomyocytes (hiPSC-CMs) offer an opportunity to better understand the pathophysiological mechanisms of the disease as well as to serve as a platform for drug screening. Here, we developed an in vitro IHD model using hiPSC-CMs in hypoxic conditions and defined the effects of the subsequent hypoxic stress on CMs functionality. Furthermore, the effect of levosimendan on hiPSC-CMs functionality was evaluated during and after hypoxic stress. The morphology, contractile, Ca^2+^-handling, and gene expression properties of hiPSC-CMs were investigated in response to hypoxia. Hypoxia resulted in significant cardiac arrhythmia and decreased Ca^2+^ transient amplitude. In addition, disorganization of sarcomere structure was observed after hypoxia induction. Interestingly, levosimendan presented significant antiarrhythmic properties, as the arrhythmia was abolished or markedly reduced with levosimendan treatment either during or after the hypoxic stress. Moreover, levosimendan presented significant protection from the sarcomere alterations induced by hypoxia. In conclusion, this chip model appears to be a suitable preclinical representation of IHD. With this hypoxia platform, detailed knowledge of the disease pathophysiology can be obtained. The antiarrhythmic effect of levosimendan was clearly observed, suggesting a possible new clinical use for the drug.

## 1. Introduction

Ischemic heart disease (IHD) is caused by inefficient or blocked blood flow to the heart due to the significant narrowing of the vessels by an intravascular plaque or an acute rupture of a plaque, resulting in reduced blood supply to the myocardium. IHD is a major cause of mortality globally [1]. Ischemia induces myocardial infarction (MI), resulting in cellular stress and cardiac dysfunction and ultimately leading to heart failure [2]. Cardiomyocytes (CMs) have a limited regeneration capability, and the lost CMs are replaced by a fibrotic scar [3]. Thus, it is important to protect the native CMs and preserve myocardial tissue against cell death in addition to the regeneration of injured heart tissue. The development of experimental models to study IHD is necessary for understanding the biological mechanisms to improve the therapeutic approaches for restoring the function of CMs injury [4].

Several studies with animal models have been conducted to address myocardial ischemia [5]. However, various physiological aspects of the human heart, such as heart rate and action potential, are different from those in animals. Additionally, animal model-based approaches are associated with ethical issues [6]. Recently, the generation of human-induced pluripotent stem cells (hiPSCs) [7] and their differentiation into cardiomyocytes (hiPSC-CMs) have offered an unprecedented opportunity to study IHD and its potential treatment options [8]. hiPSC-CMs not only recapitulate the physiology of the human heart but are also expected to reflect the sensitivity of the donor cells to various drugs [9]. Thus, hiPSC-CMs have been widely used for in vitro cardiac disease modeling, drug discovery, and cardiac toxicity screenings [10,11].

Previous studies have investigated the functionality of hiPSC-CMs in IHD modeling and illustrated the adverse effect of hypoxia on the electrophysiological parameters of hiPSC-CMs during the different stages of ischemia [9,12,13,14]. However, hiPSC-CM ischemia studies are still very limited in number, and further advanced analyses of CM functionality in response to hypoxia are needed.

Levosimendan is a Ca^2+^ enhancer belonging to a new class of positive inotropic agents with Ca^2+^-sensitizing activity; it is currently used in the treatment of heart failure [15,16,17]. Levosimendan sensitizes troponin C to Ca^2+^ by stabilizing the Ca^2+^ bound conformation of troponin and increasing the myofilaments’ sensitivity to Ca^2+^, thereby increasing the effects of Ca^2+^ on cardiac myofilaments during systole and improving contraction at a low energy cost; however, it does not affect the diastolic function [18].

In this study, the main objective was to develop an in vitro IHD model using hiPSC-CMs and define the subsequent effects of hypoxic stress on CM functionality. To address this, the morphology, the contractile, Ca^2+^-handling properties, and gene expression properties of hiPSC-CMs were investigated under hypoxic stress. The hiPSC-CMs showed structural changes, abnormalities in Ca^2+^ transients, and beating rhythm in response to hypoxia. We also evaluated the potential protective effect of levosimendan on CMs either during or after hypoxic stress, and we found a significant antiarrhythmic effect of levosimendan on ischemia-induced arrhythmia. These findings suggest that our model is a useful platform for ischemia modeling that could enhance the repurposing of old drugs as well as the development of novel therapies for IHD.

## 2. Materials and Methods

### 2.1. Generation and Differentiation of hiPSC Lines

This study was carried out in accordance with the guidelines of the Ethics Committee of Pirkanmaa Hospital District for establishing, culturing, and differentiating hiPSC lines (Aalto-Setälä R08070). A skin biopsy for hiPSC establishment was received from the Heart Hospital, Tampere University Hospital, Finland. Both written and verbal information about the study were provided, and informed consent was given. The hiPSC line UTA.04602.WT used in this study was generated from the dermal fibroblasts of a healthy 55-year-old female [19]. The derivation of the cell line was carried out by reprogramming the fibroblasts using the retroviral vectors *OCT4*, *KLF4*, c-*MYC*, and *SOX2*, as previously described [7]. hiPSCs were differentiated into CMs by two methods, the mouse endoderm-like cell line (END-2) co-culture method [20] and the small-molecule differentiation method [21], which both formed beating CM aggregates. We did some parallel experiments by using both of the differentiation methods to avoid method-related bias. The beating CM aggregates were dissociated mechanically and enzymatically with collagenase A (Roche Diagnostics) to a single-cell level 30 to 60 days from the beginning of the differentiation, as described earlier [22]. Dissociated CMs were plated on a 0.17 mm coverslip on the glass base of the OxyGenie 1-well chamber [12,23,24], coated with 0.1% gelatin at either (1) a high density suited to form a confluent synchronously beating monolayer or (2) a low density to retain the single-cell level for further analysis. Dissociated CMs were cultured with embryoid body formation medium (EB medium), which composed of FluoroBrite™ DMEM (Gibco) supplemented with 20% knockout serum replacement (Gibco), 1% NEAA (Gibco), 1% GlutaMAX (Gibco), and 0.5% penicillin/streptomycin (Lonza); the medium was changed every other day until the day of the experiment. Dissociated CMs were cultured in a humidified incubator at 37 °C and 5% CO_2_ until the day of the experiment. In the levosimendan (Sigma-Aldrich) experiments, the drug was solubilized into dimethyl sulfoxide (DMSO; Life Technologies) to a stock concentration of 10 mM and further diluted in EB medium to a final concentration of 2 µM.

### 2.2. Hypoxia Setup

**Figure 1:**
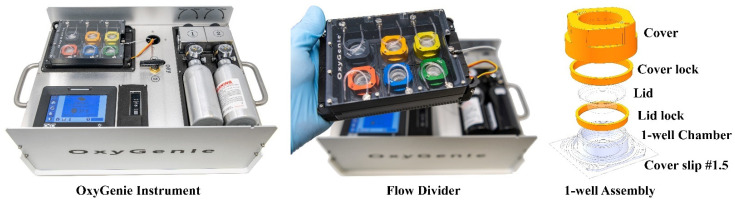
Structure and function of the OxyGenie mini-incubator: 1-well assembly includes a 1-well chamber on a glass plate, a lid and lid lock, and a cover and cover lock to maintain the required gas environment.

Hypoxia induction was performed using a novel portable system called the OxyGenie mini-incubator (The Baker Company, Sanford, ME, USA), as described earlier [12,24]. Briefly, the OxyGenie is a portable platform that provides a controlled environment for prolonged hypoxia/normoxia studies outside a traditional incubator, e.g., during Ca^2+^ or video imaging. The OxyGenie mini-incubator includes a battery-operated temperature controller with two prefilled and replaceable gas cylinders, an indium tin oxide (ITO) heater plate, a custom-made 6-well flow divider, and 1–6 individual 1-well culture chambers made from silicone elastomer with covers and lids (Figure 1). Gas is supplied from the refillable gas bottle filled with premixed gas. In this study, premixed gas with a composition of 1% O_2_, 5% CO_2_, and 94% N_2_ (Oy Linde Gas Ab, Espoo, Finland) was used for hypoxia induction. The gas was supplied at 5 mL/min to the chamber assembly.

### 2.3. Experimental Setup

**Figure 2:**
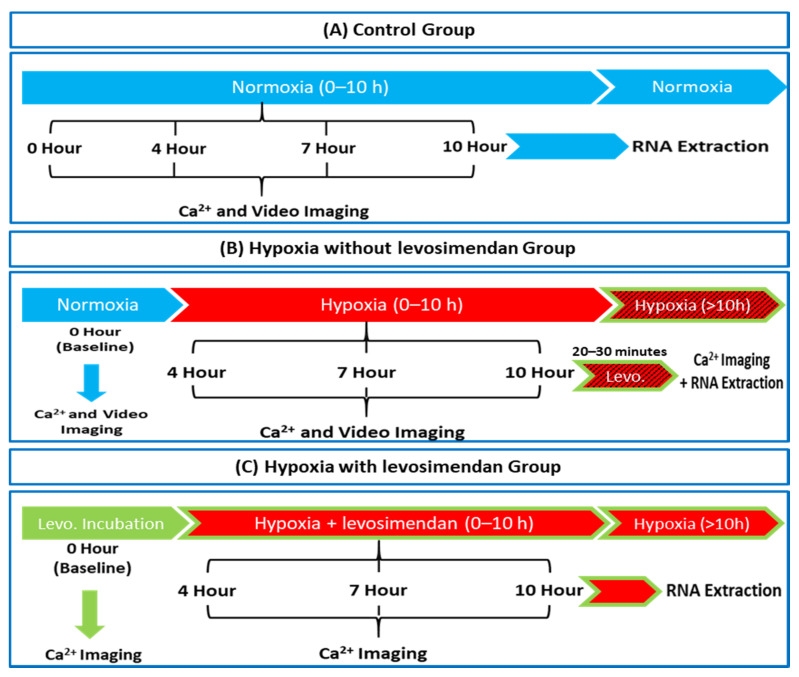
Schematic diagram of the experimental workflow in (**A**) control, (**B**) hypoxia without levosimendan (levo.), and (**C**) hypoxia with levosimendan groups.

Ca^2+^ and video imaging was performed 4 to 5 days after cell dissociation. In the Ca^2+^ imaging experiment, the setup consisted of three groups: control, hypoxia without levosimendan, and hypoxia with levosimendan. In the video imaging experiment, the setup consisted of two groups: control and hypoxia without levosimendan. In the control group, CMs were cultured in EB medium in a pre-warmed OxyGenie mini-incubator (37 °C) in normoxia conditions, using 19% O_2_ and 5% CO_2_ throughout the experimental period. In the hypoxia without levosimendan group, CMs were loaded into a pre-warmed OxyGenie mini-incubator (37 °C), and hypoxia was initiated using 1% O_2_ and 5% CO_2_ (hypoxic) gas for 10 h. After 10 h, the levosimendan was injected inside the chambers through the silicone layer using a syringe to avoid the disturbance of the hypoxic condition (only with Ca^2+^ imaging samples). Ca^2+^ imaging was performed after levosimendan incubation for 20 to 30 min in hypoxic conditions. In the hypoxia with levosimendan group, CMs were first incubated with the levosimendan for 20 to 30 min in a pre-warmed OxyGenie mini-incubator (37 °C) in normoxia conditions using 19% O_2_ and 5% CO_2_; then, hypoxia was initiated using 1% O_2_ and 5% CO_2_ (hypoxic) gas for 10 h. From all groups, RNA samples were collected after Ca^2+^ imaging. The time periods used for imaging were 0 h (baseline), 4 h, 7 h, and 10 h in all different groups. A confluent monolayer and single dissociated hiPSC-CMs were both used in the experiment, and the data were pooled together due to the similar parameter values in both the monolayer and single cells. An experimental workflow of the Ca^2+^ and video imaging of all the treatment groups is presented in Figure 2.

### 2.4. Ca^2+^ Imaging

The Ca^2+^ indicator Cal-520^®^ AM (Cat. no. 21130; AAT Bioquest) was used to record the Ca^2+^ transient of hiPSC-CMs. Cal-520^®^ AM was dissolved into DMSO to obtain a 5 mM stock solution, which was further diluted with extracellular solution to a final concentration of 5 µM on the day of the experiment. The extracellular solution consisted of (in mM) 137 NaCl, 5 KCl, 0.44 KH_2_PO_4_, 20 HEPES, 4.2 NaHCO_3_, 5 D-glucose, 2 CaCl_2_, 1.2 MgCl_2_, and 1 Na-pyruvate, and the pH was adjusted to 7.4 with NaOH. CMs were loaded with Cal-520^®^ AM for 90 min at 37 °C, after which the indicator was washed off and replaced with fresh extracellular solution for a 30 min de-esterification process before starting the Ca^2+^ imaging. Ca^2+^ imaging was performed at a controlled temperature of 37 °C. The Ca^2+^ fluorescence signals were imaged with an Axio Observer A1 microscope with a Fluar 20x/0.75 M27 Objective (Carl Zeiss Microscopy GmbH, Jena, Germany). Images were acquired with an Andor iXon3 885 EM-CCD camera (Andor Technology, Belfast, Northern Ireland) synchronized with a Lambda DG-4 Plus (Sutter Instrument, Novato, CA, USA) wavelength switcher with the Zeiss filter set 69 (Carl Zeiss Microscopy GmbH, Jena, Germany). The imaging software Zen 2.3 blue edition (Carl Zeiss Microscopy GmbH, Jena, Germany) was used to control the light source and camera during recording. Cal-520^®^ AM was excited at 480 nm, the emission was measured at 540 nm, and time-series image sequences were collected at 30 frames per second. For the Ca^2+^ imaging analysis, single beating CMs or a monolayer of beating CMs were selected as regions of interest (ROI), and background fluorescence recorded from a cell-free area was subtracted before further data processing occurred. The recorded Ca^2+^ traces were further analyzed with the Clampfit software version 10.5 (Molecular Devices, Silicon Valley, CA, USA).

### 2.5. Video Microscopy Imaging

Videos of spontaneously beating CMs were recorded by using a microscope (Nikon Eclipse TS100, Tokyo, Japan) with an Optika DIGI-12 video camera (Optika Microscopes, Ponteranica, Italy). Videos were recorded for 30 s at 60 frames per second, and the temperature was controlled at 37 °C. To quantify contractions, a beating rhythm analysis was performed with the open-source software tool MUSCLEMOTION on the ImageJ software according to the manufacturer’s instructions [25]. The recorded beating rhythms were further analyzed with the Clampfit software version 10.5 (Molecular Devices, Silicon Valley, CA, USA).

### 2.6. Immunocytochemistry

In control samples, dissociated CMs were washed with twice 1× PBS and fixed in 4% paraformaldehyde (PFA, Sigma-Aldrich) for 20 min. In the hypoxia without levosimendan samples, CMs were transferred to hypoxic conditions using 1% O_2_ and 5% CO_2_ (hypoxic) gas for 20 h. Subsequently, levosimendan was injected inside the chambers of some samples for 2 h after hypoxia. In the hypoxia with levosimendan group, CMs were first incubated with the levosimendan for 20 to 30 min in a pre-warmed OxyGenie mini-incubator (37 °C) in normoxic conditions using 19% O_2_ and 5% CO_2_; then, hypoxia was initiated using 1% O_2_ and 5% CO_2_ (hypoxic) gas for 20 h. After hypoxia, cells were washed once with 1× PBS and fixed directly in 4% paraformaldehyde. Subsequently, the cells were blocked with a blocking solution (10% NDS, 0.1% TritonX-100, 1% BSA in PBS) for 45 min. Then, the cells were incubated with mouse monoclonal Anti-α-Actinin (1:1500; Sigma-Aldrich; A7811) and rabbit anti-HIF1α (1:500; Invitrogen; 700505) as primary antibodies overnight at 4 °C. After washing three times with 1% BSA in PBS, cells were incubated subsequently with donkey anti-mouse Alexa Fluor 568 (1:1000; Invitrogen; A10037) and polyclonal swine anti-rabbit IgG HRP (1:2000; Dako; P0217) as secondary antibodies for 1 h incubation in room temperature in the dark. Finally, the cell nuclei were stained using Vectashield mounting medium with DAPI (Vector Laboratories). The fluorescence images were acquired using an Olympus IX51 fluorescence microscope.

### 2.7. Reverse Transcription Polymerase Chain Reaction (RT-PCR)

The total RNA of CMs was extracted after Ca^2+^ imaging using a Monarch^®^ Total RNA Miniprep Kit following the manufacturer’s protocol. RNA concentration and quality were measured using a NanoDrop spectrophotometer (NanoDrop Technologies) before further analysis. The reverse transcription of the total RNA to cDNA was performed using a High-Capacity cDNA Reverse Transcription Kit (Applied Biosystems) following the manufacturer’s instructions. qPCR for cDNA samples was performed using the TaqMan Gene Expression Master Mix (Applied Biosystems) following the manufacturer’s instructions and according to the standard protocols for an ABI7300 thermal cycler (Applied Biosystems). Three technical replicates were used for each sample in each assay. TaqMan assays for *RYR2*, *ATP2A2*, *SLC8A1*, *CACNA1C*, *TNNT2*, *HIF1A*, *ABCC9*, KCNJ8, and KCNJ11 were carried out, and GAPDH served as a housekeeping gene (Table S2). The fold expression changes were calculated using the 2^−ΔΔCT^ method, as described previously [26].

### 2.8. Statistical Analysis

Statistical analysis was carried out with IBM SPSS Statistics 26 (SPSS, Chicago, IL, USA). In the Ca^2+^ and video imaging analysis, comparisons within the same group (at different time points) were performed with a nonparametric Wilcoxon signed ranks test and between the different groups (at the same time point) with a nonparametric Kruskal–Wallis test followed by a Tukey HSD post-hoc test. An independent samples Kruskal–Wallis test was used for the analysis of qPCR samples. Statistical significance levels were *p* < 0.05 (*), *p* < 0.01 (**), and *p* < 0.001 (***). The data are presented as means ± standard deviations or the standard error of the mean (SEM).

## 3. Results

### 3.1. Ca^2+^ Handling of hiPSC-CMs and the Effect of Levosimendan

**Figure 3:**
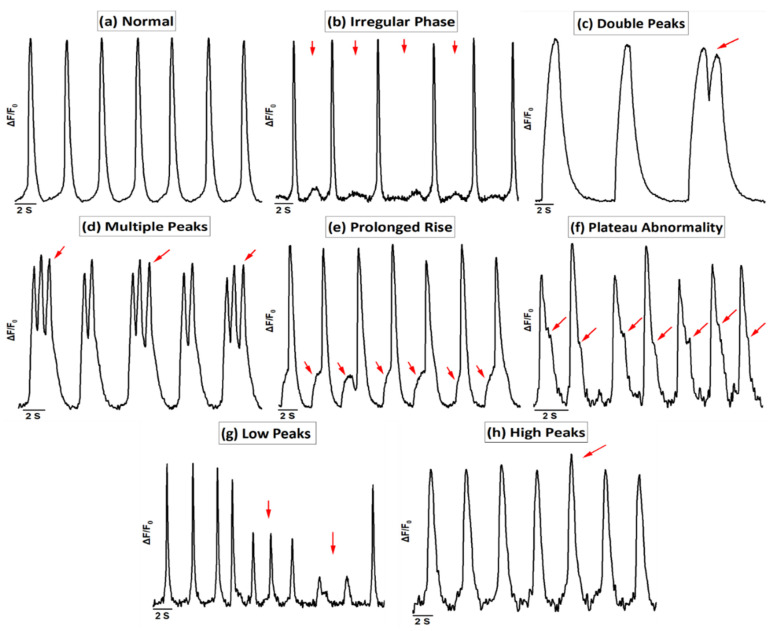
Representative Ca^2+^ transient categories of hiPSC-CMs. (**a**) Normal. Abnormal Ca^2+^ traces showing (**b**) irregular phase, (**c**) double peaks, (**d**) multiple peaks, (**e**) prolonged rise, (**f**) plateau abnormality, (**g**) low peaks, and (**h**) high peaks.

In the Ca^2+^ imaging, the hiPSC-CMs showed abnormal Ca^2+^ transients under hypoxia that were categorized into the following groups: irregular phase, double peaks, multiple peaks, prolonged rise, plateau abnormality, low peaks, and high peaks (Figure 3). Ca^2+^ traces were determined as normal (N) if the rhythm of the hiPSC-CMs was regular, in which case the phases of contraction and relaxation followed each other without any delay or additional movement (Figure 3a; Online Movie S1). Any deviations from that were considered abnormal traces. Abnormal Ca^2+^ transients were categorized as follows: irregular phase (IP), if there were unequal intervals between the Ca^2+^ transients (Figure 3b; Online Movie S2); double peaks (DP), if there were two successive peaks in the same transient without decaying to the baseline (Figure 3c; Online Movie S3); multiple peaks (MP), if there were three or more successive peaks in the same transient without decaying to the baseline (Figure 3d; Online Movie S4); prolonged rise (PR), if the rise time of the Ca^2+^ transient was prolonged by displaying a notch in the rise transient (Figure 3e; Online Movie S5); plateau abnormality (PA), if the decay time of the Ca^2+^ transient was prolonged by displaying a notch in the decay transient (Figure 3f; Online Movie S6); low peaks (LP), if the amplitude of any transient was 10%–90% of the amplitude of a normal peak in the trace (Figure 3g; Online Movie S7); and high peaks (HP), if the amplitude of any transient was 10% higher than the amplitude of a normal peak in the trace (Figure 3h; Online Movie S8).

**Figure 4:**
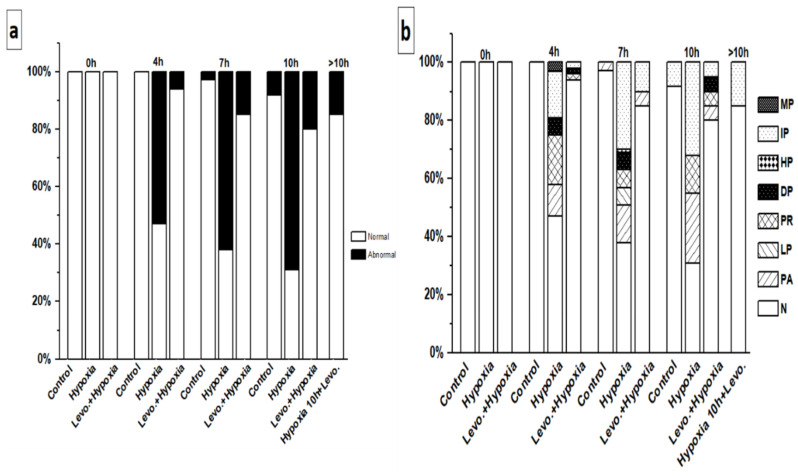
Incidence percent of (**a**) normal and abnormal Ca^2+^ transients of hiPSC-CMs. (**b**) Different Ca^2+^ transient abnormality categories in control, hypoxia without levosimendan, and hypoxia with levosimendan groups. The sample sizes in control group were 0 h (baseline) *n* = 38, 4 h *n* = 34, 7 h *n* = 36, and 10 h *n* = 37; in the hypoxia without levosimendan group, the sample size was 0 h (baseline) *n* = 46, 4 h *n* = 57, 7 h *n* = 56, 10 h *n* = 53 and following 10 h *n* = 20; and in the hypoxia with levosimendan group, the sample size was 0 h (baseline) *n* = 19, 4 h *n* = 24, 7 h *n* = 19, and 10 h *n* = 18.

**Figure 5:**
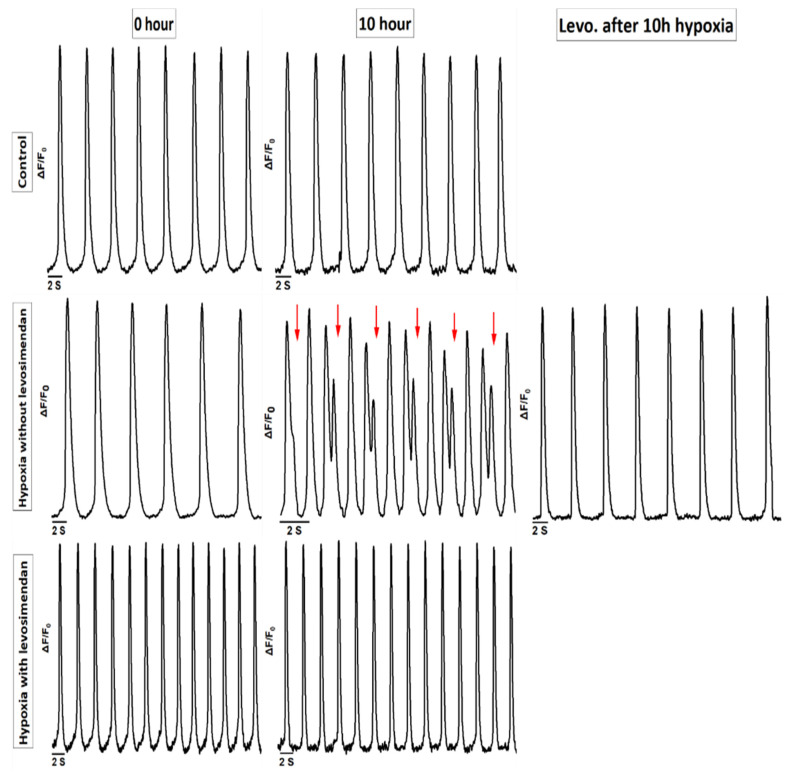
Levosimendan addition abolished or significantly reduced ischemia-induced arrhythmia either during or after hypoxic stress.

In the control group, hiPSC-CMs showed a regular Ca^2+^ transient until 4 h. However, after 7 and 10 h, 3% and 8% of the hiPSC-CMs, respectively, showed Ca^2+^ transient abnormalities (Figure 4a). In the hypoxia without levosimendan group, hiPSC-CMs showed regular Ca^2+^ transients at the baseline, but hypoxia induction caused a substantial increase in Ca^2+^ transient abnormalities, and 53%, 62%, and 69% of the hiPSC-CMs showed an abnormal Ca^2+^ transient after 4, 7, and 10 h of hypoxia, respectively. With levosimendan addition after 10 h of hypoxia, the percentage of hiPSC-CMs abnormalities decreased from 69% to 15% (Figure 4a). If the cells were cultured in the presence of levosimendan before hypoxia induction, arrhythmias were significantly decreased, and the percentages of the arrhythmias were 6%, 15%, and 20% after 4, 7, and 10 h of hypoxia, respectively (Figure 4a and Figure 5).

The types of Ca^2+^ transient abnormalities varied between the different groups. In the control group at 7 h, the detected abnormalities were plateau abnormality (3%), and at 10 h, they were irregular phase (8%) (Figure 4b). In the hypoxia without levosimendan group at 4 h, the detected abnormalities were prolonged rise, irregular phase, plateau abnormality, double peaks, and multiple peaks in 17%, 16%, 11%, 6%, and 3% of the hiPSC-CMs, respectively (53% in total). At 7 h, the abnormalities were irregular phase, plateau abnormality, low peaks, double peaks, prolonged rise, and high peak in 30%, 13%, 6%, 6%, 6%, and 1% of the hiPSC-CMs, respectively (62% in total). At 10 h, the abnormalities were irregular phase, plateau abnormality, and prolonged rise in 32%, 24%, and 13% of the hiPSC-CMs, respectively (69% in total). After 10 h of hypoxia, the addition of levosimendan decreased the percentage of abnormalities significantly, and the remaining abnormalities were irregular phase (15%) (Figure 4b). In the hypoxia with levosimendan group after 4 h, the detected abnormalities were double peaks, prolonged rise, and irregular phase, each found in 2% of the hiPSC-CMs. At 7 h, the abnormalities were irregular phase and plateau abnormality in 10% and 5% of the hiPSC-CMs, respectively. At 10 h, the abnormalities were double peaks, plateau abnormality, prolonged rise, and irregular phase, each found in 5% of the hiPSC-CMs (Figure 4b).

**Figure 6:**
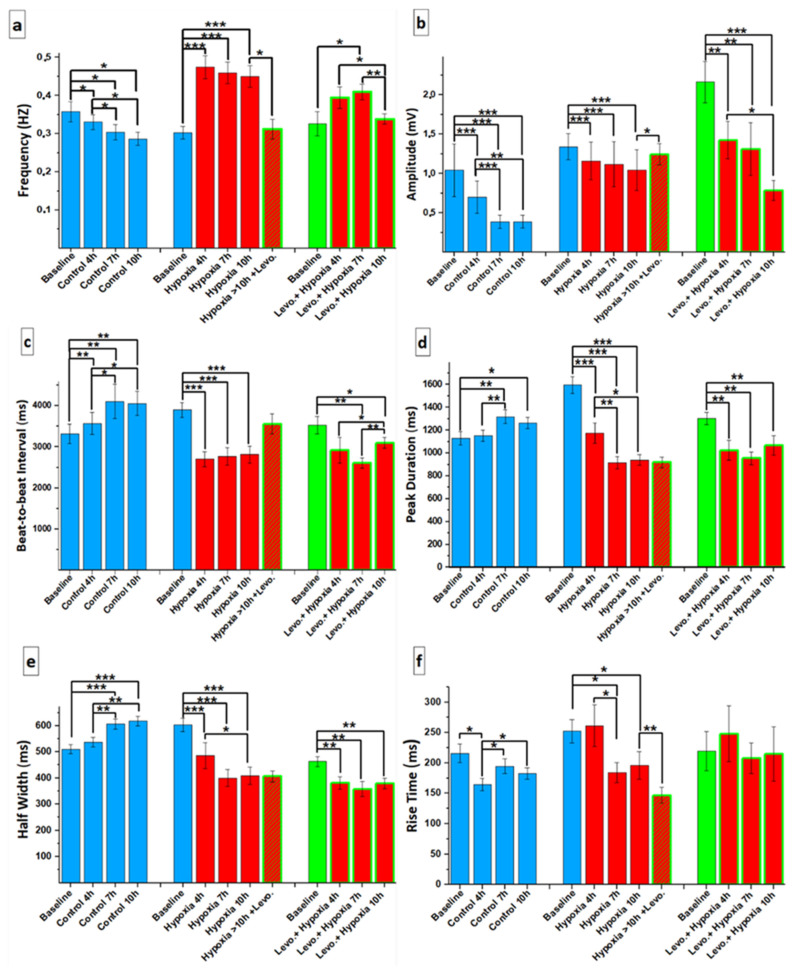

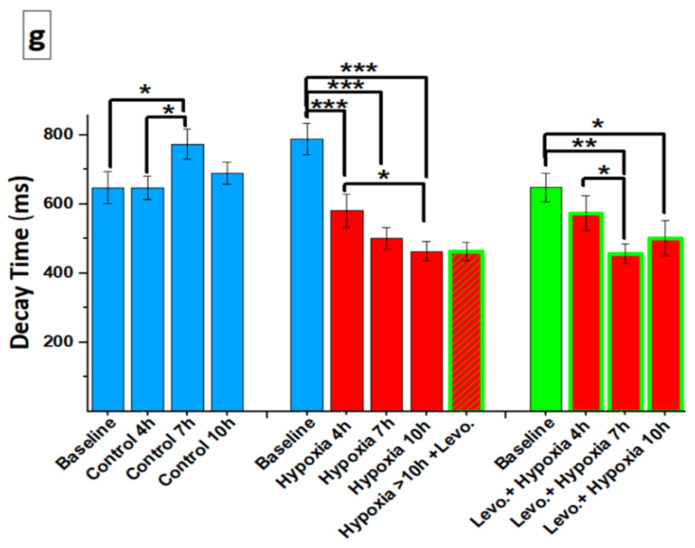
Parameters of the Ca^2+^ transient in the control and hypoxic conditions with or without levosimendan until 10 h and after levosimendan addition following 10 h of hypoxia: (**a**) frequency: the beating frequency of CMs; (**b**) ΔF/F0: Ca^2+^ peak amplitude; (**c**) beat-to-beat interval: time from the beginning of one Ca^2+^ transient until the beginning of the next transient; (**d**) peak duration: Ca^2+^ peak duration; (**e**) half-width: duration at half-maximum amplitude; (**f**) rise time 10% to 90%: rise time of the Ca^2+^ transient starting from 10% until 90% of the transient rise; (**g**) decay time 90% to 10%: decay time of the Ca^2+^ transient starting from 90% until 10% of the transient decay. Data are shown as averages. Statistical significance: * *p* < 0.05; ** *p* < 0.01; and *** *p* < 0.001. Error bars represent the standard error of the mean (SEM).

The hiPSC-CMs showed an increased beating rate under hypoxic conditions with or without levosimendan when compared to the baseline in the normoxic condition. The addition of levosimendan after 10 h of hypoxia decreased the frequency, approaching the values of the baseline before hypoxia induction (Figure 6a). In addition, the hiPSC-CMs also showed a significant decrease in the Ca^2+^ transient amplitude and beat-to-beat interval values under hypoxic conditions with or without levosimendan when compared to the baseline in the normoxic condition. These values also started to increase after adding levosimendan following 10 h of hypoxia, resembling the values observed in the baseline before hypoxia induction (Figure 6b,c). The peak duration and half-width of the Ca^2+^ transients were greatly decreased under hypoxic conditions with or without levosimendan compared to the baseline in the normoxic condition. The addition of levosimendan after 10 h of hypoxia did not have an effect on these parameters (Figure 6d,e). Moreover, the rise time of Ca^2+^ transients was greatly decreased under hypoxic conditions without levosimendan compared to the baseline in the normoxic condition, and it was still decreased after adding levosimendan following 10 h of hypoxia (Figure 6f). In addition, the decay time was greatly decreased under hypoxic conditions with or without levosimendan compared to the baseline in the normoxic condition, and it started to increase slightly after adding levosimendan following 10 h of hypoxia (Figure 6g). We added a supplementary table showing some readouts, which were performed in cells derived from both of the differentiation methods “mouse endoderm-like cell line (END-2) co-culture method and small-molecule differentiation method” (Table S1).

### 3.2. Beating Characteristics of hiPSC-CMs with Video Imaging

In the video imaging, the hiPSC-CMs showed an abnormal beating rhythm under hypoxia, and these abnormalities resembled those observed with Ca^2+^ imaging (Figure S1 and Online Movie S9–S16).

**Figure 7:**
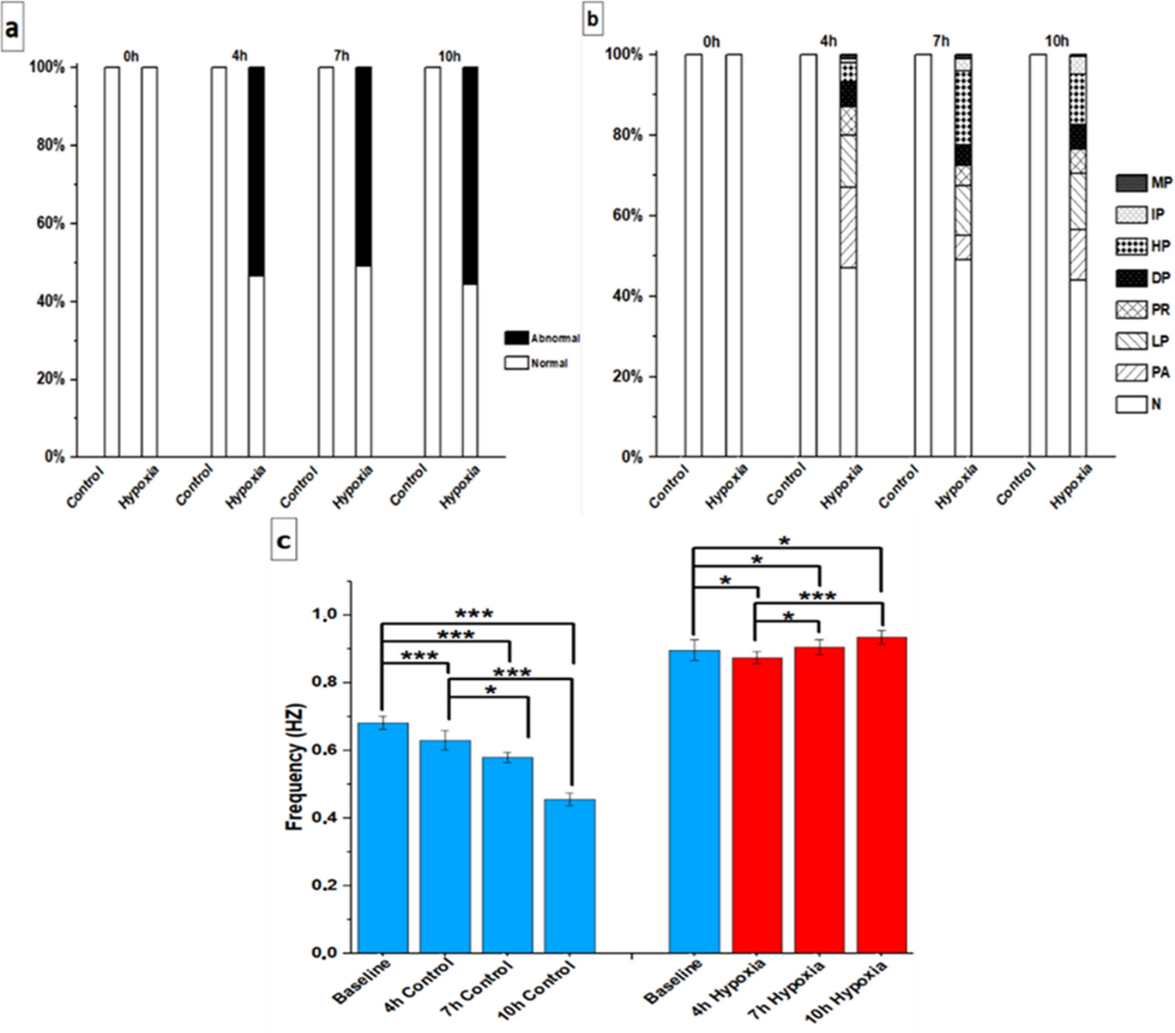
(**a**) Incidence percent of normal and abnormal rhythm of hiPSC-CMs and (**b**) incidence percent of different rhythm abnormality categories. (**c**) Beating frequency of hiPSC-CMs in control and hypoxia without levosimendan groups. The sample sizes in the control group were 0 h (baseline) *n* = 38, 4 h *n* = 36, 7 h *n* = 35, and 10 h *n* = 38 and under the hypoxic condition were 0 h (baseline) *n* = 20, 4 h *n* = 76, 7 h *n* = 112, and 10 h *n* = 110. Data are shown as averages. Statistical significance: * *p* < 0.05 and *** *p* < 0.001. Error bars represent the standard error of the mean (SEM).

In the control group, all hiPSC-CMs were found to be beating normally at all different time points. In the hypoxia without levosimendan group, hiPSC-CMs were categorized as normal at the baseline; however, the rhythm abnormalities increased considerably after hypoxia induction. The abnormality percentages were 53%, 51%, and 56% after 4, 7, and 10 h of hypoxia, respectively (Figure 7a). The beating rhythm categories varied between the groups. After 4 h of hypoxia, there were different types of abnormalities, including plateau abnormality, low peaks, prolonged rise, double peaks, high peaks, irregular phase, and multiple peaks in 20%, 13%, 7%, 6%, 5%, 1%, and 1%, of the hiPSC-CMs, respectively (53% in total). At 7 h, the abnormalities were high peaks, low peaks, plateau abnormality, prolonged rise, double peaks, irregular phase, and multiple peaks in 18%, 13%, 6%, 5%, 5%, 3%, and 1% of the hiPSC-CMs, respectively (51% in total). At 10 h, the abnormalities were low peaks, high peaks, plateau abnormality, prolonged rise, double peaks, irregular phase, and multiple peaks in 14%, 12%, 12%, 6%, 6%, 5%, and 1% of the hiPSC-CMs, respectively (56% in total) (Figure 7b).

In the control group, hiPSC-CMs showed a reduction in frequency rate within the different timepoints. In the hypoxia without levosimendan group, hiPSC-CMs showed a reduction in frequency rate after 4 h of hypoxia, which started to increase at 7 h and 10 h of hypoxia when compared to the baseline in the normoxic condition (Figure 7c).

### 3.3. Immunocytochemistry

**Figure 8:**
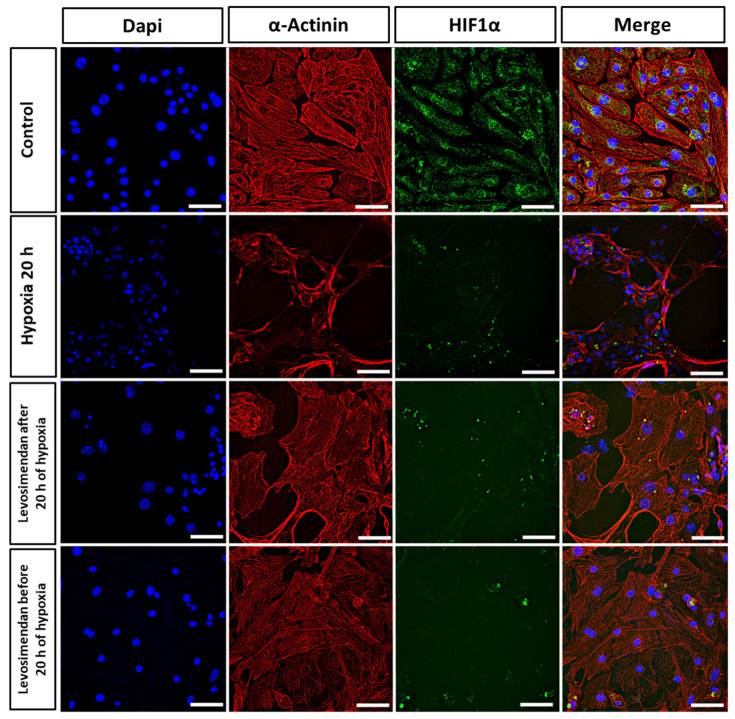
Representative images of immunocytochemical staining of hiPSC-CMs by cardiac alpha-actinin (red), HIF1α (green), and dapi (blue). Hypoxia changed the structure, sarcomere organization, and nucleus size of hiPSC-CMs. Levosimendan addition abolished or significantly reduced the structure and sarcomere alteration either during or after hypoxic stress. Scale bar corresponds to 20 µm.

By immunofluorescent staining, we investigated the sarcomere structure and organization (α-Actinin, red), nuclei size (DAPI, blue), and HIF1α expression (HIF1α, green) of the hiPSC-CMs in control and hypoxic conditions. Moreover, we tested the effect of levosimendan addition on hypoxic CMs. In the control conditions, the cells were spreading properly through the plate, without alteration in either sarcomere structure or organization, and the HIF1α expression was expressed and distributed well through the cytoplasm (Figure 8). After hypoxia induction, we observed dramatic sarcomere disorganization in the majority of hiPSC-CMs, and the cell structure seems to deteriorate after 20 h of hypoxia. Furthermore, the size of the nuclei seems to decrease with a reduction in the expression of HIF1α. With the addition of levosimendan after 20 h of hypoxia, the cells did not show deteriorations in sarcomere structure in the majority of hiPSC-CMs and also increased expression of HIF1A. Furthermore, the nuclei increased in size when compared to hypoxic cells. Similarly, if levosimendan was present in cell culture media during the whole duration of ischemia, the cells were spreading properly through the plate, without alterations in the sarcomere organization in the majority of hiPSC-CMs. Furthermore, the nuclei size was larger when compared to hypoxic cells, and the nuclei size resembled that of the control cells (Figure 8).

### 3.4. Gene Expression Analysis

**Figure 9:**
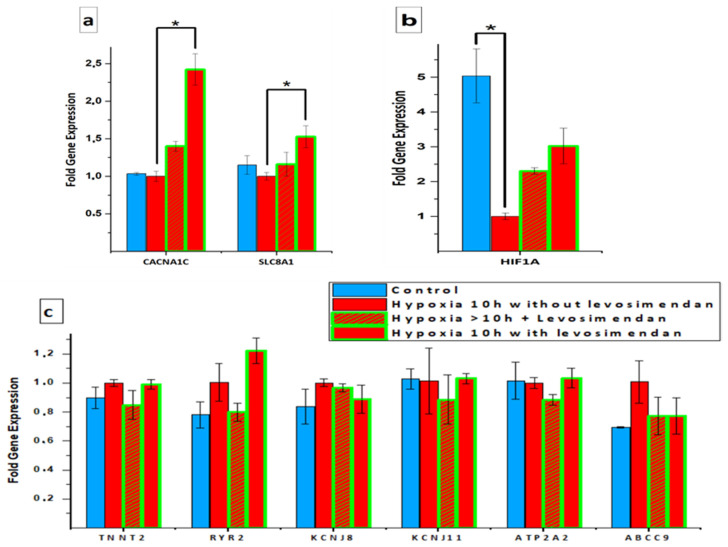
Relative gene expression levels of cardiac-related genes: (**a**) Ca^2+^-handling (*CACNA1C* and *SLC8A1*) gene expression; (**b**) hypoxia marker (*HIF1A*) gene expression; (**c**) *RYR2*, *ATP2A2*, *TNNT2*, *ABCC9*, *KCNJ8*, and *KCNJ11* gene expression. Samples from qPCR analysis presented as mean + standard deviation. * Statistical significance (*p* < 0.05).

qPCR was performed to compare the expression of certain genes in hiPSC-CMs in the control, hypoxia without levosimendan, and hypoxia with levosimendan groups. A statistically significant increase was observed in the expression of the *CACNA1C* and *SLC8A1* genes, which encode Ca^2+^-handling proteins, in the hypoxia with levosimendan group when compared to the hypoxia without levosimendan group. In the hypoxia without levosimendan group, the mean expressions of *CACNA1C* and *SLC8A1* were 1.00 ± 0.07- and 1.00 ± 0.05-fold, respectively. A slight but not statistically significant increase was also observed in the expression of *CACNA1C* in the hypoxia without levosimendan group after levosimendan incubation (1.40 ± 0.06-fold increase). In the levosimendan group with hypoxia, the mean expressions of *CACNA1C* and *SLC8A1* were significantly increased by 2.42 ± 0.20- and 1.53 ± 0.15-fold, respectively (*p* < 0.05, Figure 9a). In addition, a statistically significant decrease was observed in the expression of a hypoxia marker (*HIF1A*) in CMs after 10 h of hypoxia (*p* < 0.05). The addition of levosimendan after 10 h of hypoxia increased the expression of *HIF1A* slightly but not statistically significantly. Similarly, if levosimendan was present in cell culture media during the whole duration of ischemia, a non-significant increase in the expression of *HIF1A* was observed (Figure 9b). There were no statistically significant differences in the expression of *RYR2*, *ATP2A2*, *TNNT2*, *ABCC9*, *KCNJ8*, and *KCNJ11* genes (Figure 9c).

## 4. Discussion

The objective of this research was to develop an in vitro IHD model using hiPSC-CMs that could be used to investigate the effects of hypoxia on CMs. The main findings were the following: (1) hypoxia was associated with abnormal Ca^2+^ transients and an abnormal beating rhythm; (2) hypoxia reduced the amplitude, peak duration, and rise and decay times of the ca^2+^ transient; (3) hypoxia changed the structure, sarcomere organization and the nuclei size of hiPSC-CMs; (4) levosimendan had significant antiarrhythmic properties, either during or after hypoxic stress; (5) levosimendan addition abolished or significantly reduced the structure and sarcomere alterations, either during or after hypoxic stress; and (6) levosimendan upregulated the expression of the *CACNA1C* and *SLC8A1* genes, which encode Ca^2+^-handling proteins, and the *HIF1A* gene, which encodes a hypoxia marker.

Preserving viability and preventing arrhythmias are key principles in the management of patients with ischemic heart disease [27]. Many currently used inotropic drugs improve the contractility of CMs by increasing intracellular concentrations of free Ca^2+^; however, they also increase the myocardial consumption of energy and even produce arrhythmias. Therefore, they are not optimal pathophysiological drugs [28,29]. Levosimendan has been introduced into clinical usage as a unique calcium enhancer with Ca^2+^-sensitizing inotropic activity [15,16,17,29]. In contrast to other inotropic agents, levosimendan exerts no effect on the Ca^2+^ concentration within the cardiac myofilaments and is therefore not associated with increases in myocardial oxygen consumption or intracellular Ca^2+^ concentration, which are known to exert potentially deleterious effects in the setting of heart failure [18]. Additionally, levosimendan does not impair the relaxation and diastolic function of CMs [30].

In this study, we demonstrated that the induction of hypoxia initially resulted in an increased frequency of Ca^2+^ transients, followed by a gradual decrease in this frequency. This observation was confirmed by video imaging with a different set of cells. An increase in beating rate in hypoxic conditions has also been reported previously in isolated embryonic chick hearts [31], embryonic rat cardiac myocytes [32], and hiPSC-CMs [14]. This likely occurs due to the changes in Na^+^ current in the heart [32]. It has been demonstrated that a very slowly inactivating persistent Na^+^ (I_Na_-P) current is enhanced during hypoxia [33,34,35,36], while a transient Na^+^ (I_Na_-T) current is reduced during hypoxia [36]. This I_Na_-P current contributes to Na^+^ loading and subsequent Ca^2+^ overload and arrhythmias [33,37]. This could explain the observed increase in beat frequency. Furthermore, Ca^2+^ overload can contribute to adenosine triphosphate (ATP) depletion via the activation of Ca^2+^-dependent ATPases [35]. This reduction in ATP formation would subsequently lead to the opening of ATP-dependent potassium (KATP) channels and thus a reduction in the mechanical activity of the myocytes [32]. This could explain the gradual frequency reduction that occurred after the initial elevation.

In contrast to the present findings, previous investigations have also reported that CM beating frequency decreased in response to ischemia [12,38,39]. This discrepancy may be due to the different medium conditions used in different studies. The culture medium composition can be modified in several ways, e.g., by serum and glucose removal [12,38,39] or acidosis [38,39,40], to better mimic the physiological ischemic event. In this study, the culture medium composition included standard cell culture medium with glucose and serum to reveal the effect of hypoxia on hiPSC-CMs without any additional stress on the CMs. In addition, other studies that used the normal standard cell culture medium with glucose and serum showed an initial elevation in the beating frequency of CMs during ischemic stress, followed by a gradual reduction later [31,32]. This indicates that the medium composition plays a role in the ischemic effect on CMs, and further studies are needed for clarification.

Hypoxia decreased both the peak and half-width durations, and this matched the elevation of the Ca^2+^ transient frequency. Furthermore, hypoxia decreased the amplitude of the Ca^2+^ transient. Our findings are in line with other studies reporting a declining amplitude in response to hypoxia [14,41]. However, the Ca^2+^ amplitude also decreased in the control and hypoxia with levosimendan groups. This could be due to a potential rapid photobleaching problem with fluorescent dyes [42], which can severely limit the recording duration over which accurate estimates of intracellular Ca^2+^ signals can be obtained [43,44]. The addition of levosimendan following 10 h of hypoxia decreased the Ca^2+^ transient frequency and increased the transient amplitude to values similar to those observed at the baseline before hypoxia induction. The changes in the frequency and the amplitude after levosimendan induction could be due to its inotropic activity [15,16]. In more detail, the administration of levosimendan leads to an opening of the active sites of troponin C, increasing its sensitivity to Ca^2+^. This would suggest that hearts treated with levosimendan should be capable of performing more work under normoxic and ischemic conditions without consuming additional ATP [16]. Moreover, levosimendan has anti-ischemic effects [18,45,46,47] through the activation of cardiac ATP-sensitive potassium channels.

Furthermore, a decrease in the beat-to-beat interval and rise and decay times in response to hypoxia was shown, most likely due to an increase in the beating/Ca^2+^ transient frequency. Our findings are in line with other studies reporting declining rise and decay times of transients in response to hypoxia [41]. A previous study reported that hypoxia was associated with a decrease in ATP levels and suggested that the hiPSC-CMs were not able to fully adapt to the hypoxic challenge and maintain their ATP levels through decreases in contractile force and oxygen consumption. Therefore, impaired ATP production may affect the systolic and relaxation function in hypoxic conditions [41]. This could explain the decrease in the Ca^2+^ transient amplitude, beat-to-beat interval, and rise and decay times in response to hypoxia. The addition of levosimendan following 10 h of hypoxia resulted in an increase in the beat-to-beat interval and decay time. This could be due to the cardioprotective actions of levosimendan, which opens ATP-sensitive potassium channels in smooth muscle cells and myocytes and allows their hyperpolarization, causing vasodilation and other cardioprotective actions [18,48].

Interestingly, in addition to the changes in the parameters of Ca^2+^ transients, the hiPSC-CMs showed abnormalities in beating rhythm. These findings are in line with a previous study that reported irregular Ca^2+^ transients in hypoxia [14]. To our knowledge, the categorization of Ca^2+^ transient and mechanical beating behavior abnormalities using video recording under hypoxic stress has not been reported together in any previous reports. Previous studies have demonstrated that ischemia induces the reverse mode mechanism of the Na^+^/Ca^2+^ exchanger (NCX) on the plasma membrane, which is responsible for elevating the cytosolic Ca^2+^ concentration, causing arrhythmia and cell death by apoptosis [28,49]. In addition, a previous study using an hiPSC-CMs model reported a surge decrease in ATP content in response to hypoxia [14,28]. As a result, the Na^+^/K^+^ pump, the main Na^+^ extrusion pathway, comes to a halt, leading to the accumulation of Na^+^ in the cytosol. A higher intracellular Na^+^ concentration, in turn, reverses the direction of action of plasma membrane NCX, causing Ca^2+^ influx and elevating the cytosolic Ca^2+^ concentration. Moreover, the CM plasma membrane and its ion channels lose their original stability, which increases intracellular Ca^2+^ and is accompanied by the rapid generation of reactive oxygen species (ROS). ROS can disturb cellular homeostasis, especially in cardiac L-type Ca^2+^ channels, inducing intracellular Ca^2+^ overload [14]. Elevating cytosolic Ca^2+^ concentration causes arrhythmia and cell death by apoptosis [28,49]. Therefore, the inhibition of reverse mode NCX activity, with reduced cellular Ca^2+^ accumulation, has been proven to provide cardioprotective action [49]. This could explain the increased abnormalities in the Ca^2+^ transient and beating rhythm of CMs seen in the current study.

An important new finding in our study is that ischemia-induced arrhythmia can be abolished or markedly reduced with levosimendan treatment either during or after the hypoxic stress. Levosimendan reduced the severe Ca^2+^ transient abnormalities, and the remaining abnormalities were limited to irregular-phase abnormality, which is considered as the mildest abnormality type and is sometimes manifested in healthy control hiPSC-CMs. This suggests a potent protective effect of levosimendan against hypoxic stress. The addition of levosimendan has been previously shown to abolish NCX activity and reduce cytosolic Ca^2+^ concentration and cellular apoptosis [49]. Therefore, the translocation of NCX away from the CM plasma membrane with levosimendan may partly explain the decreased NCX activity. This could explain the antiarrhythmic property of levosimendan in the current study, although the clinical findings have been contradictory [50,51]. To the best of our knowledge, no previous reports have confirmed the antiarrhythmic property of levosimendan before our results.

Dramatic changes were observed in hiPSC-CM morphology, sarcomere structure, and nucleus size after hypoxia induction. There was a clear disruption and disorganization in the sarcomere structures of hiPSC-CMs after hypoxic stress. Moreover, the size of the nuclei in the hiPSC-CMs decreased after hypoxic stress. Our findings are in line with other studies that have reported the same changes in the sarcomere structure and nucleus size in response to hypoxia [12]. Furthermore, although *HIF1A* is known to play a crucial role in the activation of hypoxia [52], it was not highly expressed in the hypoxia condition in our results. This also matches with our results in gene expression in this study, as we revealed that *HIF1A* was not highly expressed in the hypoxia condition. In addition, our findings are in line with other studies that have reported a non-significant increase in the expression of the *HIF1A* gene in response to hypoxia [12]. The structural disorganization and the changes in the nucleus size are correlated to cell apoptosis [53,54]. Interestingly, levosimendan addition abolished or significantly reduced the structure and sarcomere deterioration in the majority of hiPSC-CMs, either during or after hypoxia. In addition, the nuclei increased in size when compared to hypoxic cells and resembling the nuclei size of the control cells. This could be because levosimendan inhibits interleukin (IL)-1β-induced apoptosis via the activation of phosphatidylinositol 3 kinase (PI3K)/protein kinase B (AKT) pathway and the inhibition of inducible nitric oxide (NO) expression in the cardiac fibroblasts [55].

In previous investigations, hypoxia was shown to affect the cardiac gene expression of hiPSC-CMs under hypoxic stress, e.g., the genes associated with the mitochondrial proton-transporting ATP synthase complex and cardiac muscle contraction [56,57]. In the current study, there were no significant changes in the expression of the *SLC8A1*, *RYR2*, and *ATP2A2* genes related to Ca^2+^-handling and the *TNNT2* gene related to the sarcomere in response to hypoxia. Our findings are in line with other studies that have reported non-significant changes in the expression of *RYR2*, *ATP2A2*, and *TNNT2* genes in response to hypoxia [41]. Furthermore, although *HIF1A* is known to play a crucial role in the activation of hypoxia [52], it was not highly expressed in the hypoxia condition in our results. Our findings are in line with other studies that have reported non-significant changes in the expression of the *HIF1A* gene in response to hypoxia [12]. It is possible that oxygen-independent *HIF1A* degradation pathways are activated [58]. Furthermore, it may be difficult to see the *HIF1A* peak at the gene expression level since it is regulated more at the protein level by the effect of oxygen [59]. Thus, we assume that *HIF1A* protein expression should be analyzed. Moreover, it is possible that the time window for collecting PCR samples was not optimal to find *HIF1A* at its peak since *HIF1A* has different kinds of downregulation pathways that are activated if the hypoxia is prolonged [59]. However, hypoxia with levosimendan resulted in the upregulated expression of the *HIF1A* gene. Our findings are in line with other studies that have reported a significant increase in the expression of the *HIF1A* gene after levosimendan treatment [60,61]. In addition, a significant increase in the expression of the *CACNA1C* and *SLC8A1* genes encoding Ca^2+^-handling proteins was observed in the hypoxia with levosimendan group when compared to the hypoxia without levosimendan group. This could be due to the inotropic activity of levosimendan [15,16]. Although levosimendan activates cardiac ATP-sensitive potassium channels [18,48], there were no significant changes in the expression of the *ABCC9*, *KCNJ8*, and *KCNJ11* genes related to potassium (KATP) channels in our results.

## 5. Conclusions

Hypoxia resulted in a significant cardiac arrhythmia with abnormal Ca^2+^ transients, irregular beating rhythm, and induced sarcomere disorganization in the CMs. Interestingly, levosimendan presented significant antiarrhythmic properties, as arrhythmias were abolished or markedly reduced with levosimendan treatment, either during or after the hypoxic stress. Moreover, levosimendan addition abolished or significantly reduced the sarcomere disorientation, either during or after hypoxic stress. These findings suggest that our model may provide a useful platform for ischemia modeling and could speed up the development of novel therapies for ischemic heart disease.

## Supplementary Materials

The following supporting information can be downloaded at: www.mdpi.com/article/10.3390/cells11061045/s1, Figure S1: Representative beating rhythm categories of hiPSC-CMs (a) normal beating rhythm showing regular rhythm. Abnormal beating rhythm showing (b) irregular phase, (c) double peaks, (d) multiple peaks, (e) prolonged rise, (f) plateau abnormality, (g) low peaks, and (h) high peak. Table S1: Representative measurements of Ca^2+^ transient Parameters, which were performed in cells derived from both differentiation methods used in our study. Table S2: TaqMan 20 × Assays used in qPCR. Online Movie S1 Control-normal Ca transient. Online Movie S2: Hypoxia-abnormal Ca transient-irregular phase. Online Movie S3: Hypoxia-abnormal Ca transient-double peaks. Online Movie S4: Hypoxia-abnormal Ca transient-multiple peaks. Online Movie S5: Hypoxia-abnormal Ca transient-prolonged rise. Online Movie S6: Hypoxia-abnormal Ca transient-plateau abnormality. Online Movie S7: Hypoxia-abnormal Ca transient-low peaks. Online Movie S8: Hypoxia-abnormal Ca transient-high peak. Online Movie S9: Control-normal beating rhythm. Online Movie S10: Hypoxia-abnormal beating rhythm-irregular phase. Online Movie S11: Hypoxia-abnormal beating rhythm-double peaks. Online Movie S12: Hypoxia-abnormal beating rhythm-multiple peaks. Online Movie S13: Hypoxia-abnormal beating rhythm-prolonged rise. Online Movie S14: Hypoxia-abnormal beating rhythm-plateau abnormality. Online Movie S15: Hypoxia-abnormal beating rhythm-low peaks. Online Movie S16: Hypoxia-abnormal beating rhythm-high peak.

## Study Limitations

In this study, we did not differentiate between CM subtypes (atrial, nodal, or ventricular). In addition, based on the results, it could be hypothesized that the hiPSC-CMs were immature and structurally, functionally, and metabolically resemble fetal more than adult CMs. They mainly use glycolysis for energy production in vitro as opposed to the predominantly oxidative metabolism observed in normal adult hearts. In addition, we did not measure the metabolic profile of the hiPSC-CMs to figure out the metabolic state of the CMs. Moreover, the Ca^2+^ and video imaging were recorded from different cells randomly, as we mostly failed to follow the same cells at all different time points, which could cause the absence of potentially interesting data. Furthermore, the slow speed of the decrease in the oxygen level in the culture, which gives the cells time to adjust to the conditions, differs from clinical conditions where acute ischemic events are typically sudden.
