# Supplementary material for: Cardiac Ischemia On-a-Chip: Antiarrhythmic Effect of Levosimendan on Ischemic Human-Induced Pluripotent Stem Cell-Derived Cardiomyocytes
Source: Cells. 2022 Mar 19;11(6):1045. doi: 10.3390/cells11061045 (PMC8947267; doi:10.3390/cells11061045)
Supplement: Supplementary file 1 [file cells-11-01045-s001.zip › cells-1604501-supplementary.v6. update.pdf]

cells

MDPI

Suplementry Data

Article

Cardiac Ischemia on-a-Chip: Antiarrhythmic Effect of Levosimendan on Ischemic Human-Induced Pluripotent Stem Cell-Derived Cardiomyocytes

Mahmoud Gaballah [1,2,*], Kirsi Penttinen [1], Joose Kreutzer [3], Antti-Juhana Mäki [3], Pasi Kallio [3] and Katriina Aalto-Setälä [1,4]

[1] Heart Group, Faculty of Medicine and Health Technology, Tampere University, 33520 Tampere, Finland; kirsi.penttinen@outlook.com (K.P.); katriina.aalto-setala@tuni.fi (K.A.-S.)
[2] Department of Forensic Medicine and Toxicology, Faculty of Veterinary Medicine, University of Sadat City, 32897 Sadat, Egypt
[3] Micro- and Nanosystems Research Group, Faculty of Medicine and Health Technology, Tampere University, 33520 Tampere, Finland; joose.kreutzer@tuni.fi (J.K.); antti-juhana.maki@tuni.fi (A.-J.M.); pasi.kallio@tuni.fi (P.K.)
[4] Heart Hospital, Tampere University Hospital, 33520 Tampere, Finland
* Correspondence: mahmoud.gaballah@tuni.fi; Tel.: +358-402574148

Ca^{2+} transient Parameters

		Frequency (Hz)			BPM (Beating per minute)		
Differentiation method	**n**	**Control**	**Hypoxia**	**Levosimendan**	**Control**	**Hypoxia**	**Levosimendan**
**END-2 co-culture method**	56	.29	.45	.34	18	27	21
**Small-molecule method**	25	.35	.49	.41	21	29	25
		**ΔF/F0 (mV)**			**Beat-to-beat Interval (ms)**		
Differentiation method	**n**	**Control**	**Hypoxia**	**Levosimendan**	**Control**	**Hypoxia**	**Levosimendan**
**END-2 co-culture method**	56	1.34	1.04	0.78	3894.61	2816.86	3095.70
**Small-molecule method**	25	0.26	0.15	0.10	3171.48	2918.25	2970.13
		**Peak duration (ms)**			**Half width (ms)**		
Differentiation method	**n**	**Control**	**Hypoxia**	**Levosimendan**	**Control**	**Hypoxia**	**Levosimendan**
**END-2 co-culture method**	56	1596.25	939.26	1065.47	602.97	408.68	379.11
**Small-molecule method**	25	1798.67	703.2	1235.29	858.07	570.83	490.59
		**Rise time 10% to 90% (ms)**			**Decay time 10% to 90% (ms)**		
Differentiation method	**n**	**Control**	**Hypoxia**	**Levosimendan**	**Control**	**Hypoxia**	**Levosimendan**
**END-2 co-culture method**	56	252.19	195.96	214.78	788.33	462.63	501.47
**Small-molecule method**	25	326.9305	264.08	286.47	899.29	740.62	790.49

Table S1: Representative measurements of Ca^{2+} transient Parameters, which were performed in cells derived from both differentiation methods "mouse endoderm-like cell line (END-2) co-culture method and the small-molecule differentiation method".

Beating characteristics of hiPSC-CMs with video imaging

The beating rhythm was determined as normal (N) if the rhythm was regular, in which case the phases of contraction and relaxation followed each other without any delay or additional movement of the cell (Figure S1a; Online Movie S9). Any deviations from that were considered as abnormal rhythm. Abnormal rhythm were categorized as follows: irregular phase (IP), if there were unequal intervals between the contraction and relaxation phases (Figure S1b; Online Movie S10); double peaks (DP), if there were two successive peaks in the same beating phase without decaying to the baseline (Figure S1c; Online Movie S11); multiple peaks (MP), if there were three or more successive peaks in the same beating phase without decaying to the baseline (Figure S1d; Online Movie S12); prolonged rise (PR), if the contraction rise time was prolonged by displaying a notch in the rise phase (Figure S1e; Online Movie S13); plateau abnormality (PA), if the relaxation decay time was prolonged by displaying a notch in the decay phase (Figure S1f; Online Movie S14); low peaks (LP), if the amplitude of any beating phases was 10-90% of the amplitude of a normal peak in the trace (Figure S1g; Online Movie S15); and high peaks (HP), if the amplitude of any beating phases was 10% higher than the amplitude of a normal peak in the trace (Figure S1h; Online Movie S16).

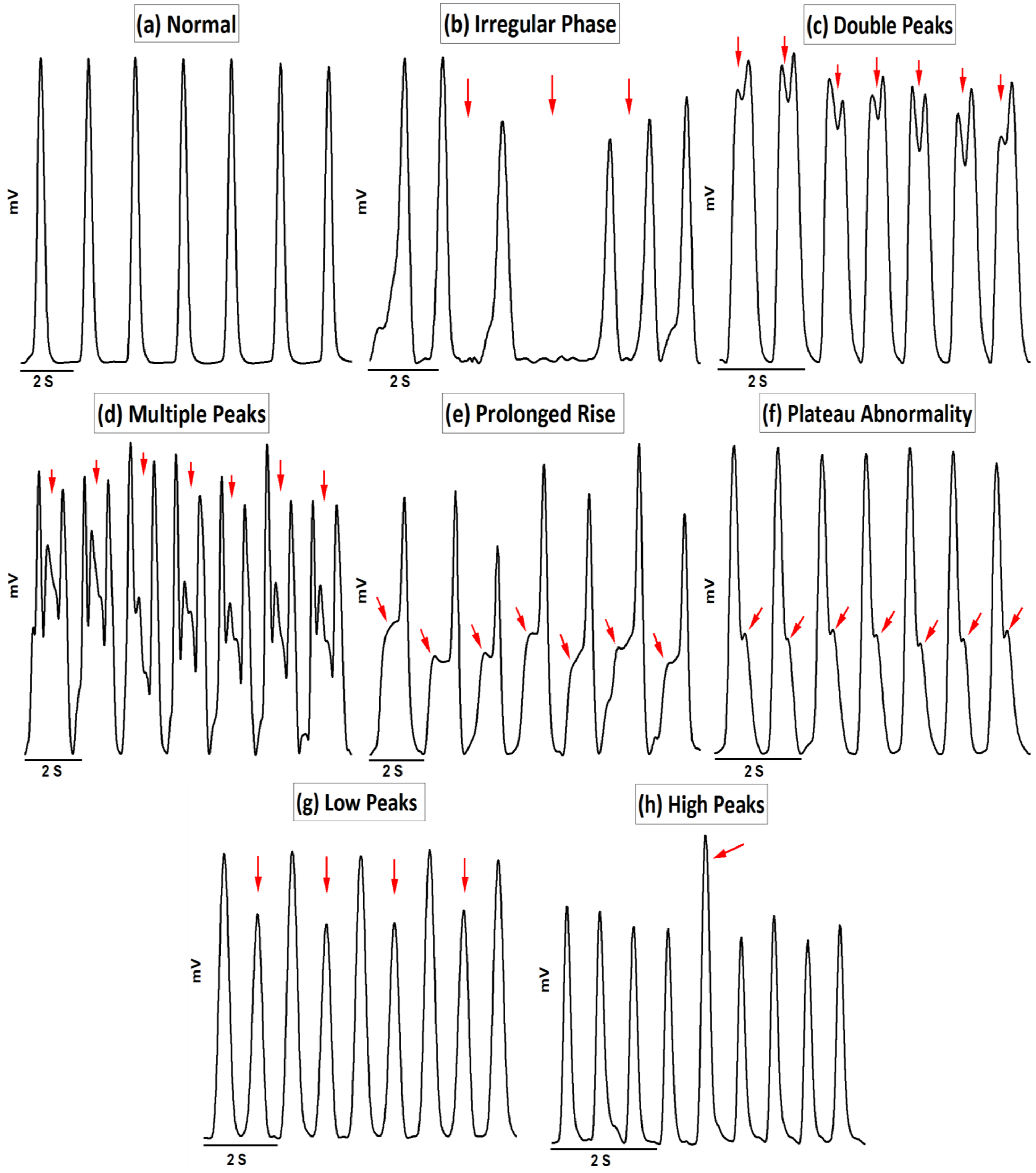

Figure S1: Representative beating rhythm categories of hiPSC-CMs. (a) Normal beating rhythm showing regular rhythm. Abnormal beating rhythm showing (b) irregular phase, (c) double peaks, (d) multiple peaks, (e) prolonged rise, (f) plateau abnormality, (g) low peaks and (h) high peak

Gene expression analysis

Table S2: TaqMan 20 × Assays used in qPCR.

Gene	Description	Function	TaqMan assay ID
GAPDH	Glyceraldehyde-3-phosphate dehydrogenase	Housekeeping gene	Hs02758991_g1
RYR2	Ryanodine receptor 2, cardiac	Ca^{2+} handling	Hs00892883_m1
ATP2A2	ATPase, calcium transporting, cardiac muscle, slow twitch 2/ SERCA2a	Ca^{2+} handling	Hs00544877_m1
SLC8A1	Solute carrier family 8, member 1/NCX1	Ca^{2+} handling	Hs01062258_m1
CACNA1C	calcium voltage-gated channel subunit alpha1 C	Ca^{2+} handling	Hs00167681_m1
TNNT2	Cardiac type troponin T2	Sarcomeric	Hs00165960_m1
HIF1A	Hypoxia inducible factor 1 alpha	Hypoxia marker	Hs00153153_m1
ABCC9	ATP binding cassette subfamily C member 9	ATP-sensitive potassium (KATP) channels	Hs00245832_m1
KCNJ8	potassium voltage-gated channel subfamily J member 8	ATP-sensitive potassium (KATP) channels	Hs00958961_m1
KCNJ11	potassium voltage-gated channel subfamily J member 11	ATP-sensitive potassium (KATP) channels	Hs00265026_s1

Online Movie Legends

Online Movie S1: Control-Normal Ca transient.

Online Movie S2: Hypoxia-Abnormal Ca transient-Irregular Phase.

Online Movie S3: Hypoxia-Abnormal Ca transient-Double peaks.

Online Movie S4: Hypoxia-Abnormal Ca transient-Multiple peaks.

Online Movie S5: Hypoxia-Abnormal Ca transient-Prolonged Rise.

Online Movie S6: Hypoxia-Abnormal Ca transient-Plateau Abnormality.

Online Movie S7: Hypoxia-Abnormal Ca transient-Low Peaks.

Online Movie S8: Hypoxia-Abnormal Ca transient-High Peaks.

Online Movie S9: Control-Normal beating rhythm.

Online Movie S10: Hypoxia-Abnormal beating rhythm-Irregular Phase.

Online Movie S11: Hypoxia-Abnormal beating rhythm-Double Peaks.

Online Movie S12: Hypoxia-Abnormal beating rhythm-Multiple Peaks.

Online Movie S13: Hypoxia-Abnormal beating rhythm-Prolonged Rise.

Online Movie S14: Hypoxia-Abnormal beating rhythm-Plateau Abnormality.

Online Movie S15: Hypoxia-Abnormal beating rhythm-Low peaks.

Online Movie S16: Hypoxia-Abnormal beating rhythm-High Peaks.
